# Crystal structure of methyl 7-phenyl-6a,7,7a,8,9,10-hexa­hydro-6*H*,11a*H*-thio­chromeno[3,4-*b*]pyrrolizine-6a-­carbox­ylate

**DOI:** 10.1107/S2056989015014024

**Published:** 2015-07-31

**Authors:** M. P. Savithri, M. Suresh, R. Raghunathan, R. Raja, A. SubbiahPandi

**Affiliations:** a Department of Physics, Queen Mary’s College (Autonomous), Chennai 600 004, India; b Department of Organic Chemistry, University of Madras, Guindy Campus, Chennai 600 025, India; c Department of Physics, Presidency College (Autonomous), Chennai 600 005, India

**Keywords:** crystal structure, thio­chromane, pyrrolizine, thio­pyran, pyrrolidine, inversion dimers, C—H⋯O hydrogen bonds

## Abstract

In the title compound, C_22_H_23_NO_2_S, the inner pyrrolidine ring (*A*) adopts an envelope conformation with the methine C atom opposite the fused C—N bond as the flap. The thio­pyran ring (*C*) has a half-chair conformation and its mean plane is inclined to the fused benzene ring by 1.74 (11)°, and by 60.52 (11)° to the mean plane of pyrrolidine ring *A*. In the outer pyrrolidine ring (*B*), the C atom opposite the fused C—N bond is disordered [site-occupancy ratio = 0.427 (13):0.573 (13)] and both rings have envelope conformations, with the disordered C atom as the flap. The planes of the phenyl ring and the benzene ring of the thio­chromane unit are inclined to one another by 65.52 (14)°. In the crystal, mol­ecules are linked by a pair of C—H⋯O hydrogen bonds forming inversion dimers.

## Related literature   

For the biological activity of pyrrolizine derivatives, see: Raj *et al.* (2003 ▸); Atal (1978 ▸); Denny (2001 ▸); Suzuki *et al.* (1994 ▸). For a related structure, see: Ramesh *et al.* (2007 ▸).
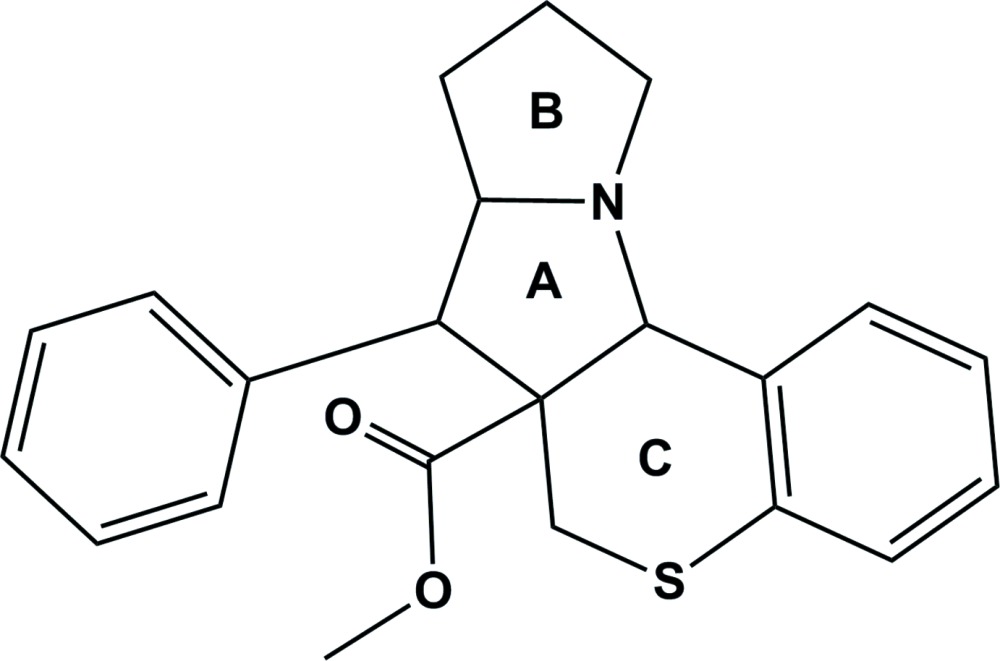


## Experimental   

### Crystal data  

C_22_H_23_NO_2_S
*M* *_r_* = 365.47
Triclinic, 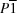

*a* = 9.5184 (4) Å
*b* = 10.4041 (5) Å
*c* = 10.6923 (4) Å
α = 81.270 (2)°
β = 66.626 (2)°
γ = 74.385 (2)°
*V* = 934.88 (7) Å^3^
*Z* = 2
Mo *K*α radiation
μ = 0.19 mm^−1^
*T* = 293 K
0.30 × 0.30 × 0.25 mm

### Data collection  

Bruker Kappa APEXII CCD diffractometer
Absorption correction: multi-scan (*SADABS*; Bruker, 2004 ▸) *T*_min_ = 0.945, *T*_max_ = 0.954
18019 measured reflections
3265 independent reflections
2504 reflections with *I* > 2σ(*I*)
*R*_int_ = 0.030

### Refinement  

*R*[*F* ^2^ > 2σ(*F* ^2^)] = 0.041
*wR*(*F* ^2^) = 0.112
*S* = 1.03
3265 reflections
246 parameters
10 restraints
H-atom parameters constrained
Δρ_max_ = 0.21 e Å^−3^
Δρ_min_ = −0.21 e Å^−3^

### 

Data collection: *APEX2* (Bruker, 2004 ▸); cell refinement: *SAINT* (Bruker, 2004 ▸); data reduction: *SAINT*; program(s) used to solve structure: *SHELXS97* (Sheldrick, 2008 ▸); program(s) used to refine structure: *SHELXL97* (Sheldrick, 2008 ▸); molecular graphics: *ORTEP-3 for Windows* (Farrugia, 2012 ▸); software used to prepare material for publication: *SHELXL97* and *PLATON* (Spek, 2009 ▸).

## Supplementary Material

Crystal structure: contains datablock(s) global, I. DOI: 10.1107/S2056989015014024/su5170sup1.cif

Structure factors: contains datablock(s) I. DOI: 10.1107/S2056989015014024/su5170Isup2.hkl

Supporting information file. DOI: 10.1107/S2056989015014024/su5170Isup3.cml

**Fig. 1.**
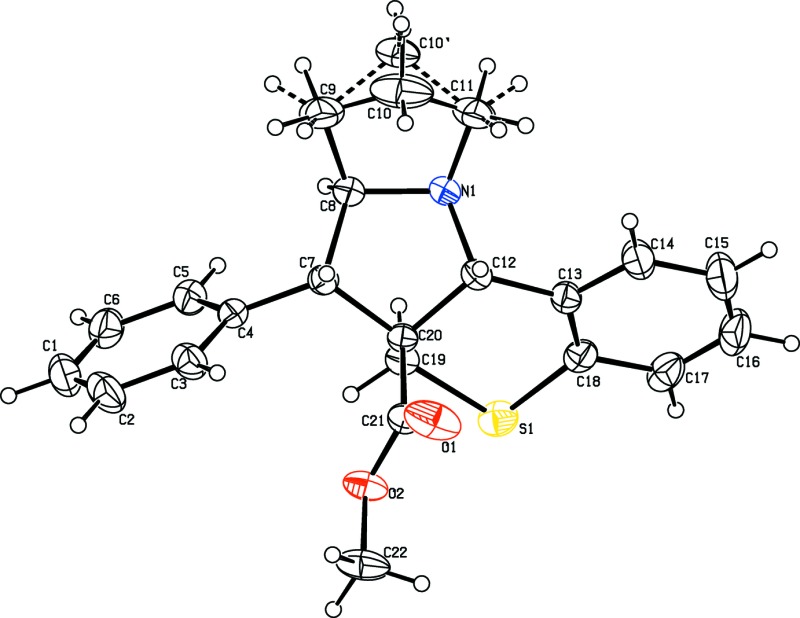
The molecular structure of the title compound, with atom labelling. Displacement ellipsoids are drawn at the 30% probability level.

**Fig. 2.**
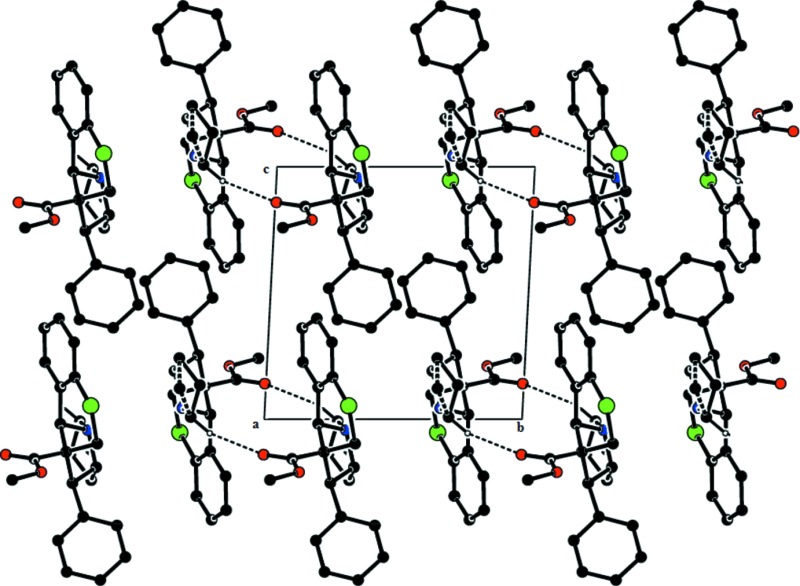
The crystal packing of the title compound, viewed along the a axis. Dashed lines shows the intermolecular C-H···O hydrogen bonds (see Table 1). H atoms not involved in hydrogen bonding have been omitted for clarity.

. DOI: 10.1107/S2056989015014024/su5170fig1.tif
The mol­ecular structure of the title compound, with atom labelling. Displacement ellipsoids are drawn at the 30% probability level.

a . DOI: 10.1107/S2056989015014024/su5170fig2.tif
The crystal packing of the title compound, viewed along the *a* axis. Dashed lines shows the inter­molecular C-H⋯O hydrogen bonds (see Table 1). H atoms not involved in hydrogen bonding have been omitted for clarity.

CCDC reference: 1414784

Additional supporting information:  crystallographic information; 3D view; checkCIF report

## Figures and Tables

**Table 1:** Hydrogen-bond geometry (, )

*D*H*A*	*D*H	H*A*	*D* *A*	*D*H*A*
C11H11*C*O1^i^	0.97	2.48	3.365(3)	151

Symmetry code: (i) 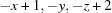.

## supplementary crystallographic information

### S1. Structural commentary

Pyrrolizidine alkaloids occur in more than 40 genera, and are responsible for heavy losses of livestock and poisoning in man due to their hepatotoxity. These alkaloids are also reported to possess a number of other biological activities (Atal, 1978) and are used as DNA minor groove alkyl­ating agents (Denny, 2001). Substituted pyrrolidines have gained much importance because they are the structural elements of many alkaloids. It has been found that they exhibit anti­fungal activity against various pathogens (Amal Raj *et al.*, 2003). Optically active pyrrolidine derivatives have been used as inter­mediates in controlled asymmetric synthesis (Suzuki *et al.*, 1994). In view of its biological importance, the crystal structure determination of the title compound was undertaken.

The molecular structure of the title compound is shown in Fig. 1. The pyrrolizine ring system is folded about the bridging N1—C8 bond, as observed in a related structure (Ramesh *et al.*, 2007). The five membered substituted pyrrolidine ring (A = N1/C7—C8/C12/C20) exhibits an envelope conformation with C20 as the flap atom [asymmetry parameter ΔCs(C20) = 1.72 (2)° and puckering parameters q_2_ = 0.463 (2)Å and φ_2_ = 288.3 (3)°]. The unsubstituted five-membered ring has a C atom disordered over two positions [site occupancies of C10 and C10' are 0.427 (13) and 0.573 (13), respectively]. The sum of bond angles around atom N1 (330°) is in accordance with sp^3^ hybridization.

In the crystal, molecules are linked by a pair of C—H···O hydrogen bonds forming inversion dimers (Table 1 and Fig. 2).

### S2. Synthesis and crystallization

A solution of methyl (*Z*)-2-(((2-formyl­phenyl)­thio)­methyl)-3-phenyl acrylate (1 mmol) and L-proline(1.2 mmol) in aceto­nitrile (10ml) was refluxed until the completion of the reaction as evidenced by TLC. The solvent was r removed under vacuum. The crude product was subjected to column chromatography on silica gel (100-200 mesh) using petroleum ether-ethyl acetate (9:1) as eluent, which successfully provided the pure product as colorless solid. The product was dissolved in chloro­form and heated for two minutes. The resulting solution was subjected to crystallization by slow evaporation of the solvent for 48 hours resulting in the formation of single crystals.

### S3. Refinement

Crystal data, data collection and structure refinement details are summarized in Table 2. All H atoms were fixed geometrically and allowed to ride on their parent C atoms: C—H = 0.93-0.98 Å with *U*_iso_(H) = 1.5*U*_eq_(C) for methyl H atoms and 1.2*U*_eq_(C) for other H atoms.

### Crystal data

**Table:**

C_22_H_23_NO_2_S	*Z* = 2
*M**_r_* = 365.47	*F*(000) = 388
Triclinic, *P*1	*D*_x_ = 1.298 Mg m^−^^3^
Hall symbol: -P 1	Mo *K*α radiation, λ = 0.71073 Å
*a* = 9.5184 (4) Å	Cell parameters from 3265 reflections
*b* = 10.4041 (5) Å	θ = 2.1–25.0°
*c* = 10.6923 (4) Å	µ = 0.19 mm^−^^1^
α = 81.270 (2)°	*T* = 293 K
β = 66.626 (2)°	Block, colourless
γ = 74.385 (2)°	0.30 × 0.30 × 0.25 mm
*V* = 934.88 (7) Å^3^	

### Data collection

**Table:**

Bruker Kappa APEXII CCD diffractometer	3265 independent reflections
Radiation source: fine-focus sealed tube	2504 reflections with *I* > 2σ(*I*)
Graphite monochromator	*R*_int_ = 0.030
ω and φ scans	θ_max_ = 25.0°, θ_min_ = 2.1°
Absorption correction: multi-scan (*SADABS*; Bruker, 2004)	*h* = −11→11
*T*_min_ = 0.945, *T*_max_ = 0.954	*k* = −12→12
18019 measured reflections	*l* = −12→12

### Refinement

**Table:**

Refinement on *F*^2^	Secondary atom site location: difference Fourier map
Least-squares matrix: full	Hydrogen site location: inferred from neighbouring sites
*R*[*F*^2^ > 2σ(*F*^2^)] = 0.041	H-atom parameters constrained
*wR*(*F*^2^) = 0.112	*w* = 1/[σ^2^(*F*_o_^2^) + (0.0453*P*)^2^ + 0.4695*P*] where *P* = (*F*_o_^2^ + 2*F*_c_^2^)/3
*S* = 1.03	(Δ/σ)_max_ = 0.001
3265 reflections	Δρ_max_ = 0.21 e Å^−^^3^
246 parameters	Δρ_min_ = −0.21 e Å^−^^3^
10 restraints	Extinction correction: *SHELXL97* (Sheldrick, 2008), Fc^*^=kFc[1+0.001xFc^2^λ^3^/sin(2θ)]^-1/4^
Primary atom site location: structure-invariant direct methods	Extinction coefficient: 0.025 (3)

### Special details

**Table:**

Geometry. All esds (except the esd in the dihedral angle between two l.s. planes) are estimated using the full covariance matrix. The cell esds are taken into account individually in the estimation of esds in distances, angles and torsion angles; correlations between esds in cell parameters are only used when they are defined by crystal symmetry. An approximate (isotropic) treatment of cell esds is used for estimating esds involving l.s. planes.
Refinement. Refinement of F^2^ against ALL reflections. The weighted R-factor wR and goodness of fit S are based on F^2^, conventional R-factors R are based on F, with F set to zero for negative F^2^. The threshold expression of F^2^ > 2sigma(F^2^) is used only for calculating R-factors(gt) etc. and is not relevant to the choice of reflections for refinement. R-factors based on F^2^ are statistically about twice as large as those based on F, and R- factors based on ALL data will be even larger.

### Fractional atomic coordinates and isotropic or equivalent isotropic displacement parameters (Å^2^)

**Table:**

	*x*	*y*	*z*	*U*_iso_*/*U*_eq_	Occ. (<1)
C1	0.9603 (3)	0.3962 (4)	0.3621 (3)	0.0705 (8)	
H1	1.0279	0.4231	0.2778	0.085*	
C2	0.9631 (3)	0.2640 (3)	0.3945 (3)	0.0702 (8)	
H2	1.0334	0.2005	0.3328	0.084*	
C3	0.8613 (3)	0.2244 (3)	0.5191 (2)	0.0541 (6)	
H3	0.8631	0.1341	0.5402	0.065*	
C4	0.7568 (2)	0.3172 (2)	0.6132 (2)	0.0417 (5)	
C5	0.7572 (3)	0.4499 (2)	0.5783 (2)	0.0535 (6)	
H5	0.6883	0.5142	0.6397	0.064*	
C6	0.8587 (3)	0.4888 (3)	0.4532 (3)	0.0655 (7)	
H6	0.8574	0.5790	0.4312	0.079*	
C7	0.6470 (2)	0.2688 (2)	0.74667 (19)	0.0383 (5)	
H7	0.6321	0.1845	0.7293	0.046*	
C8	0.4827 (2)	0.3576 (2)	0.8094 (2)	0.0488 (6)	
H8	0.4892	0.4514	0.7964	0.059*	
C9	0.3619 (3)	0.3412 (4)	0.7578 (3)	0.0890 (11)	
H9A	0.3418	0.4164	0.6965	0.107*	0.427 (13)
H9B	0.4002	0.2600	0.7085	0.107*	0.427 (13)
H9C	0.2928	0.4264	0.7482	0.107*	0.573 (13)
H9D	0.4119	0.2990	0.6710	0.107*	0.573 (13)
C10	0.2151 (9)	0.3342 (11)	0.8782 (7)	0.057 (2)	0.427 (13)
H10A	0.1624	0.2720	0.8659	0.069*	0.427 (13)
H10B	0.1432	0.4214	0.8954	0.069*	0.427 (13)
C10'	0.2769 (10)	0.2563 (10)	0.8649 (7)	0.091 (2)	0.573 (13)
H10C	0.1701	0.2721	0.8687	0.109*	0.573 (13)
H10D	0.3275	0.1631	0.8465	0.109*	0.573 (13)
C11	0.2750 (3)	0.2854 (3)	0.9919 (3)	0.0691 (8)	
H11A	0.2000	0.3268	1.0756	0.083*	0.427 (13)
H11B	0.2873	0.1894	1.0068	0.083*	0.427 (13)
H11C	0.2653	0.2084	1.0558	0.083*	0.573 (13)
H11D	0.1886	0.3601	1.0319	0.083*	0.573 (13)
C12	0.5497 (2)	0.21009 (19)	0.98166 (19)	0.0364 (5)	
H12	0.5360	0.1253	0.9643	0.044*	
C13	0.5386 (2)	0.2033 (2)	1.1270 (2)	0.0407 (5)	
C14	0.4304 (3)	0.1386 (3)	1.2261 (2)	0.0595 (6)	
H14	0.3685	0.1005	1.2005	0.071*	
C15	0.4123 (4)	0.1294 (3)	1.3608 (3)	0.0781 (8)	
H15	0.3387	0.0858	1.4254	0.094*	
C16	0.5026 (4)	0.1842 (3)	1.3991 (3)	0.0767 (9)	
H16	0.4899	0.1786	1.4905	0.092*	
C17	0.6118 (3)	0.2473 (3)	1.3047 (2)	0.0611 (7)	
H17	0.6728	0.2845	1.3323	0.073*	
C18	0.6326 (3)	0.2566 (2)	1.1671 (2)	0.0450 (5)	
C19	0.7422 (2)	0.35732 (19)	0.9027 (2)	0.0398 (5)	
H19A	0.6539	0.4339	0.9156	0.048*	
H19B	0.8314	0.3793	0.8260	0.048*	
C20	0.7012 (2)	0.23802 (18)	0.87029 (19)	0.0339 (4)	
C21	0.8360 (2)	0.1159 (2)	0.8429 (2)	0.0395 (5)	
C22	1.1091 (3)	0.0345 (3)	0.7589 (3)	0.0748 (8)	
H22A	1.2034	0.0674	0.7209	0.112*	
H22B	1.1072	−0.0189	0.6941	0.112*	
H22C	1.1068	−0.0189	0.8409	0.112*	
N1	0.42652 (18)	0.31903 (18)	0.95607 (17)	0.0428 (4)	
O1	0.8198 (2)	0.00459 (16)	0.8592 (2)	0.0818 (6)	
O2	0.97460 (16)	0.14517 (15)	0.78985 (17)	0.0542 (4)	
S1	0.78888 (7)	0.32480 (6)	1.05291 (6)	0.0536 (2)	

### Atomic displacement parameters (Å^2^)

**Table:**

	*U*^11^	*U*^22^	*U*^33^	*U*^12^	*U*^13^	*U*^23^
C1	0.0631 (17)	0.108 (2)	0.0412 (14)	−0.0378 (17)	−0.0105 (12)	0.0030 (15)
C2	0.0528 (15)	0.103 (2)	0.0478 (15)	−0.0215 (15)	−0.0017 (12)	−0.0246 (15)
C3	0.0475 (13)	0.0635 (15)	0.0458 (13)	−0.0114 (11)	−0.0091 (11)	−0.0121 (11)
C4	0.0354 (11)	0.0533 (13)	0.0355 (11)	−0.0070 (10)	−0.0147 (9)	−0.0018 (10)
C5	0.0506 (13)	0.0552 (15)	0.0470 (13)	−0.0061 (11)	−0.0162 (11)	0.0030 (11)
C6	0.0671 (17)	0.0736 (18)	0.0585 (16)	−0.0253 (15)	−0.0279 (14)	0.0176 (14)
C7	0.0363 (10)	0.0405 (11)	0.0365 (11)	−0.0051 (9)	−0.0130 (9)	−0.0056 (9)
C8	0.0350 (11)	0.0623 (15)	0.0406 (12)	−0.0016 (10)	−0.0121 (9)	−0.0007 (10)
C9	0.0467 (15)	0.157 (3)	0.0638 (18)	0.0036 (19)	−0.0299 (14)	−0.028 (2)
C10	0.035 (4)	0.066 (5)	0.074 (4)	−0.010 (3)	−0.021 (3)	−0.015 (4)
C10'	0.054 (4)	0.097 (6)	0.143 (6)	−0.006 (4)	−0.047 (4)	−0.057 (5)
C11	0.0339 (12)	0.0849 (19)	0.0797 (19)	−0.0148 (13)	−0.0153 (12)	0.0063 (15)
C12	0.0365 (10)	0.0324 (10)	0.0383 (11)	−0.0071 (8)	−0.0112 (9)	−0.0050 (8)
C13	0.0413 (11)	0.0363 (11)	0.0361 (11)	0.0000 (9)	−0.0103 (9)	−0.0053 (9)
C14	0.0585 (15)	0.0644 (16)	0.0444 (14)	−0.0149 (13)	−0.0089 (11)	0.0015 (12)
C15	0.083 (2)	0.087 (2)	0.0419 (15)	−0.0166 (17)	−0.0062 (14)	0.0051 (14)
C16	0.093 (2)	0.081 (2)	0.0349 (14)	0.0101 (17)	−0.0200 (15)	−0.0067 (13)
C17	0.0727 (17)	0.0586 (15)	0.0491 (15)	0.0103 (13)	−0.0314 (13)	−0.0163 (12)
C18	0.0486 (12)	0.0380 (12)	0.0425 (12)	0.0068 (10)	−0.0190 (10)	−0.0105 (9)
C19	0.0394 (11)	0.0328 (11)	0.0467 (12)	−0.0076 (9)	−0.0149 (9)	−0.0048 (9)
C20	0.0328 (10)	0.0310 (10)	0.0367 (10)	−0.0049 (8)	−0.0123 (8)	−0.0047 (8)
C21	0.0376 (11)	0.0351 (12)	0.0432 (12)	−0.0045 (9)	−0.0131 (9)	−0.0070 (9)
C22	0.0391 (13)	0.0678 (18)	0.108 (2)	0.0126 (12)	−0.0237 (14)	−0.0337 (16)
N1	0.0315 (9)	0.0497 (11)	0.0400 (10)	−0.0050 (8)	−0.0089 (7)	−0.0015 (8)
O1	0.0528 (10)	0.0327 (10)	0.1383 (19)	−0.0052 (8)	−0.0149 (11)	−0.0088 (10)
O2	0.0325 (8)	0.0463 (9)	0.0772 (11)	−0.0004 (7)	−0.0141 (7)	−0.0175 (8)
S1	0.0555 (4)	0.0559 (4)	0.0602 (4)	−0.0130 (3)	−0.0294 (3)	−0.0123 (3)

### Geometric parameters (Å, º)

**Table:**

C1—C6	1.360 (4)	C11—H11A	0.9700
C1—C2	1.362 (4)	C11—H11B	0.9700
C1—H1	0.9300	C11—H11C	0.9700
C2—C3	1.381 (3)	C11—H11D	0.9700
C2—H2	0.9300	C12—N1	1.476 (3)
C3—C4	1.384 (3)	C12—C13	1.507 (3)
C3—H3	0.9300	C12—C20	1.530 (3)
C4—C5	1.375 (3)	C12—H12	0.9800
C4—C7	1.505 (3)	C13—C14	1.388 (3)
C5—C6	1.381 (3)	C13—C18	1.392 (3)
C5—H5	0.9300	C14—C15	1.373 (4)
C6—H6	0.9300	C14—H14	0.9300
C7—C8	1.526 (3)	C15—C16	1.359 (4)
C7—C20	1.562 (3)	C15—H15	0.9300
C7—H7	0.9800	C16—C17	1.366 (4)
C8—N1	1.472 (3)	C16—H16	0.9300
C8—C9	1.514 (3)	C17—C18	1.396 (3)
C8—H8	0.9800	C17—H17	0.9300
C9—C10'	1.445 (8)	C18—S1	1.752 (2)
C9—C10	1.488 (7)	C19—C20	1.520 (3)
C9—H9A	0.9700	C19—S1	1.793 (2)
C9—H9B	0.9700	C19—H19A	0.9700
C9—H9C	0.9700	C19—H19B	0.9700
C9—H9D	0.9700	C20—C21	1.511 (3)
C10—C11	1.506 (7)	C21—O1	1.187 (2)
C10—H10A	0.9700	C21—O2	1.313 (2)
C10—H10B	0.9700	C22—O2	1.435 (3)
C10'—C11	1.428 (6)	C22—H22A	0.9600
C10'—H10C	0.9700	C22—H22B	0.9600
C10'—H10D	0.9700	C22—H22C	0.9600
C11—N1	1.463 (3)		
			
C6—C1—C2	119.9 (2)	N1—C11—H11A	109.7
C6—C1—H1	120.1	C10—C11—H11A	109.7
C2—C1—H1	120.1	C10'—C11—H11B	81.2
C1—C2—C3	120.0 (3)	N1—C11—H11B	109.7
C1—C2—H2	120.0	C10—C11—H11B	109.7
C3—C2—H2	120.0	H11A—C11—H11B	108.2
C2—C3—C4	121.0 (2)	C10'—C11—H11C	110.9
C2—C3—H3	119.5	N1—C11—H11C	110.9
C4—C3—H3	119.5	C10—C11—H11C	132.3
C5—C4—C3	117.9 (2)	H11A—C11—H11C	78.6
C5—C4—C7	123.32 (19)	H11B—C11—H11C	31.7
C3—C4—C7	118.8 (2)	C10'—C11—H11D	110.9
C4—C5—C6	120.9 (2)	N1—C11—H11D	110.9
C4—C5—H5	119.6	C10—C11—H11D	78.1
C6—C5—H5	119.6	H11A—C11—H11D	33.9
C1—C6—C5	120.4 (3)	H11B—C11—H11D	132.5
C1—C6—H6	119.8	H11C—C11—H11D	108.9
C5—C6—H6	119.8	N1—C12—C13	111.97 (15)
C4—C7—C8	117.60 (17)	N1—C12—C20	102.97 (15)
C4—C7—C20	117.97 (16)	C13—C12—C20	116.79 (16)
C8—C7—C20	101.70 (15)	N1—C12—H12	108.2
C4—C7—H7	106.2	C13—C12—H12	108.2
C8—C7—H7	106.2	C20—C12—H12	108.2
C20—C7—H7	106.2	C14—C13—C18	118.1 (2)
N1—C8—C9	106.7 (2)	C14—C13—C12	118.3 (2)
N1—C8—C7	105.94 (16)	C18—C13—C12	123.55 (19)
C9—C8—C7	115.6 (2)	C15—C14—C13	121.7 (3)
N1—C8—H8	109.5	C15—C14—H14	119.1
C9—C8—H8	109.5	C13—C14—H14	119.1
C7—C8—H8	109.5	C16—C15—C14	119.6 (3)
C10'—C9—C10	34.0 (3)	C16—C15—H15	120.2
C10'—C9—C8	102.1 (3)	C14—C15—H15	120.2
C10—C9—C8	107.7 (3)	C15—C16—C17	120.6 (2)
C10'—C9—H9A	139.4	C15—C16—H16	119.7
C10—C9—H9A	110.2	C17—C16—H16	119.7
C8—C9—H9A	110.2	C16—C17—C18	120.6 (3)
C10'—C9—H9B	81.7	C16—C17—H17	119.7
C10—C9—H9B	110.2	C18—C17—H17	119.7
C8—C9—H9B	110.2	C13—C18—C17	119.4 (2)
H9A—C9—H9B	108.5	C13—C18—S1	123.83 (16)
C10'—C9—H9C	111.0	C17—C18—S1	116.60 (19)
C10—C9—H9C	78.1	C20—C19—S1	111.91 (14)
C8—C9—H9C	111.4	C20—C19—H19A	109.2
H9A—C9—H9C	33.9	S1—C19—H19A	109.2
H9B—C9—H9C	132.2	C20—C19—H19B	109.2
C10'—C9—H9D	111.6	S1—C19—H19B	109.2
C10—C9—H9D	133.3	H19A—C19—H19B	107.9
C8—C9—H9D	111.3	C21—C20—C19	112.36 (16)
H9A—C9—H9D	79.2	C21—C20—C12	112.39 (16)
H9B—C9—H9D	31.5	C19—C20—C12	110.86 (16)
H9C—C9—H9D	109.2	C21—C20—C7	108.94 (15)
C9—C10—C11	102.6 (5)	C19—C20—C7	112.48 (16)
C9—C10—H10A	111.2	C12—C20—C7	99.11 (14)
C11—C10—H10A	111.2	O1—C21—O2	122.81 (19)
C9—C10—H10B	111.2	O1—C21—C20	123.89 (19)
C11—C10—H10B	111.2	O2—C21—C20	113.15 (17)
H10A—C10—H10B	109.2	O2—C22—H22A	109.5
C11—C10'—C9	108.8 (4)	O2—C22—H22B	109.5
C11—C10'—H10C	109.9	H22A—C22—H22B	109.5
C9—C10'—H10C	109.9	O2—C22—H22C	109.5
C11—C10'—H10D	109.9	H22A—C22—H22C	109.5
C9—C10'—H10D	109.9	H22B—C22—H22C	109.5
H10C—C10'—H10D	108.3	C11—N1—C8	107.08 (18)
C10'—C11—N1	104.2 (3)	C11—N1—C12	115.18 (18)
C10'—C11—C10	33.9 (3)	C8—N1—C12	108.21 (15)
N1—C11—C10	109.6 (3)	C21—O2—C22	116.65 (18)
C10'—C11—H11A	138.5	C18—S1—C19	101.43 (10)
			
C6—C1—C2—C3	0.8 (4)	C16—C17—C18—C13	1.4 (3)
C1—C2—C3—C4	−0.7 (4)	C16—C17—C18—S1	−173.83 (19)
C2—C3—C4—C5	0.2 (3)	S1—C19—C20—C21	−61.3 (2)
C2—C3—C4—C7	179.2 (2)	S1—C19—C20—C12	65.39 (18)
C3—C4—C5—C6	0.1 (3)	S1—C19—C20—C7	175.32 (13)
C7—C4—C5—C6	−178.8 (2)	N1—C12—C20—C21	−159.41 (15)
C2—C1—C6—C5	−0.4 (4)	C13—C12—C20—C21	77.5 (2)
C4—C5—C6—C1	0.0 (4)	N1—C12—C20—C19	73.91 (18)
C5—C4—C7—C8	33.6 (3)	C13—C12—C20—C19	−49.2 (2)
C3—C4—C7—C8	−145.3 (2)	N1—C12—C20—C7	−44.48 (17)
C5—C4—C7—C20	−88.8 (2)	C13—C12—C20—C7	−167.61 (16)
C3—C4—C7—C20	92.3 (2)	C4—C7—C20—C21	−68.8 (2)
C4—C7—C8—N1	−157.46 (17)	C8—C7—C20—C21	160.99 (17)
C20—C7—C8—N1	−27.0 (2)	C4—C7—C20—C19	56.5 (2)
C4—C7—C8—C9	84.7 (3)	C8—C7—C20—C19	−73.7 (2)
C20—C7—C8—C9	−144.9 (2)	C4—C7—C20—C12	173.63 (17)
N1—C8—C9—C10'	−17.6 (5)	C8—C7—C20—C12	43.42 (19)
C7—C8—C9—C10'	99.9 (5)	C19—C20—C21—O1	154.4 (2)
N1—C8—C9—C10	17.1 (6)	C12—C20—C21—O1	28.6 (3)
C7—C8—C9—C10	134.6 (5)	C7—C20—C21—O1	−80.2 (3)
C10'—C9—C10—C11	60.9 (6)	C19—C20—C21—O2	−30.0 (2)
C8—C9—C10—C11	−24.5 (7)	C12—C20—C21—O2	−155.88 (17)
C10—C9—C10'—C11	−71.8 (7)	C7—C20—C21—O2	95.31 (19)
C8—C9—C10'—C11	32.0 (7)	C10'—C11—N1—C8	21.1 (5)
C9—C10'—C11—N1	−33.9 (7)	C10—C11—N1—C8	−13.9 (5)
C9—C10'—C11—C10	70.2 (8)	C10'—C11—N1—C12	−99.3 (5)
C9—C10—C11—C10'	−62.4 (7)	C10—C11—N1—C12	−134.3 (5)
C9—C10—C11—N1	23.8 (7)	C9—C8—N1—C11	−1.9 (3)
N1—C12—C13—C14	81.6 (2)	C7—C8—N1—C11	−125.6 (2)
C20—C12—C13—C14	−160.09 (19)	C9—C8—N1—C12	122.8 (2)
N1—C12—C13—C18	−99.5 (2)	C7—C8—N1—C12	−0.9 (2)
C20—C12—C13—C18	18.9 (3)	C13—C12—N1—C11	−84.8 (2)
C18—C13—C14—C15	1.6 (3)	C20—C12—N1—C11	148.95 (19)
C12—C13—C14—C15	−179.4 (2)	C13—C12—N1—C8	155.49 (18)
C13—C14—C15—C16	−0.3 (4)	C20—C12—N1—C8	29.2 (2)
C14—C15—C16—C17	−0.5 (4)	O1—C21—O2—C22	−3.5 (3)
C15—C16—C17—C18	0.0 (4)	C20—C21—O2—C22	−179.14 (19)
C14—C13—C18—C17	−2.1 (3)	C13—C18—S1—C19	19.2 (2)
C12—C13—C18—C17	178.92 (18)	C17—C18—S1—C19	−165.83 (16)
C14—C13—C18—S1	172.73 (16)	C20—C19—S1—C18	−47.73 (16)
C12—C13—C18—S1	−6.3 (3)		

### Hydrogen-bond geometry (Å, º)

**Table:**

*D*—H···*A*	*D*—H	H···*A*	*D*···*A*	*D*—H···*A*
C11—H11*C*···O1^i^	0.97	2.48	3.365 (3)	151

Symmetry code: (i) −*x*+1, −*y*, −*z*+2.
