# Supplementary figures and images for: Crystal structure of methyl 7-phenyl-6a,7,7a,8,9,10-hexa­hydro-6H,11aH-thio­chromeno[3,4-b]pyrrolizine-6a-­carbox­ylate
Source: Acta Crystallogr E Crystallogr Commun. 2015 Jul 31;71(Pt 8):o627–8. doi: 10.1107/S2056989015014024 (PMC4571432; doi:10.1107/S2056989015014024)

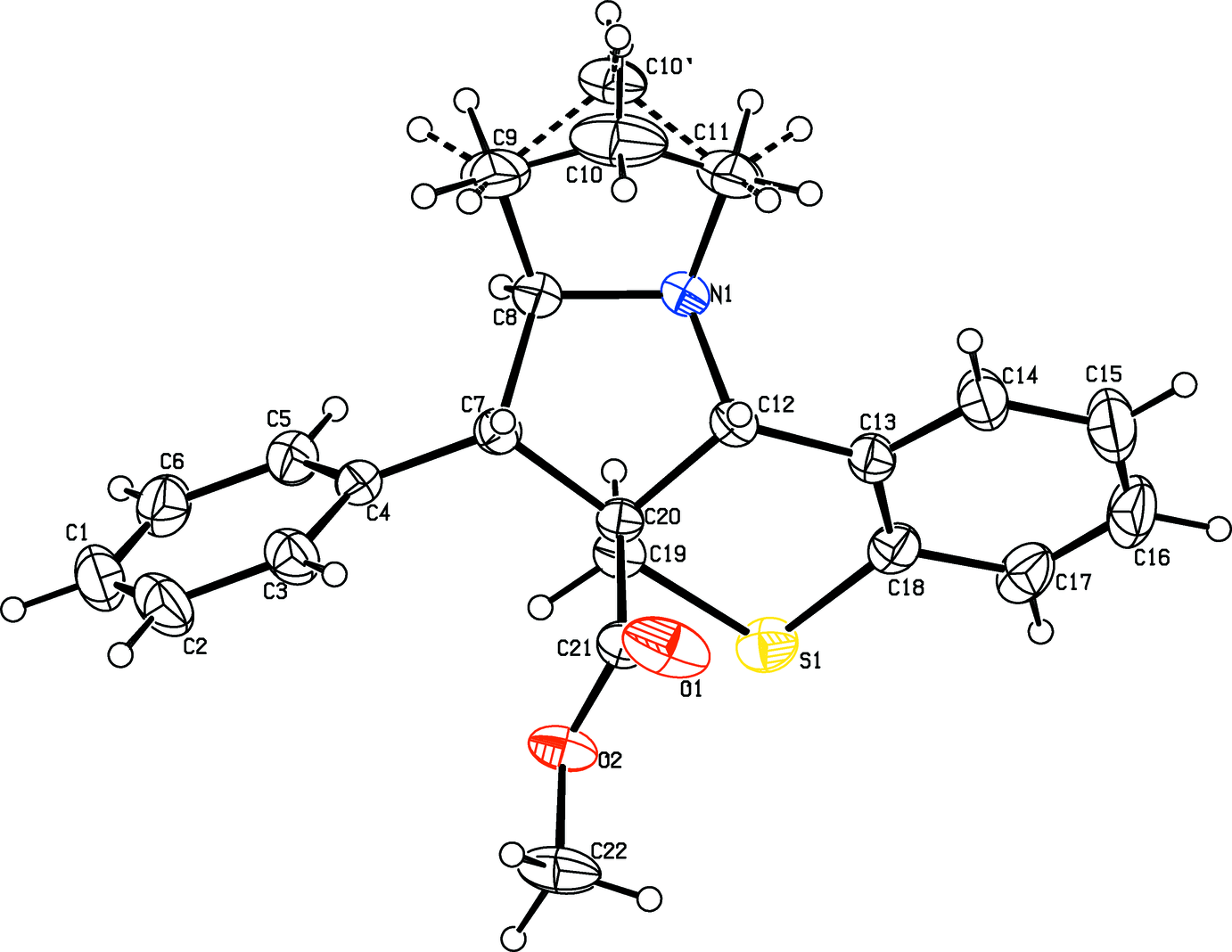

Supplement: Supplementary file 4 [file e-71-0o627-fig1.tif]

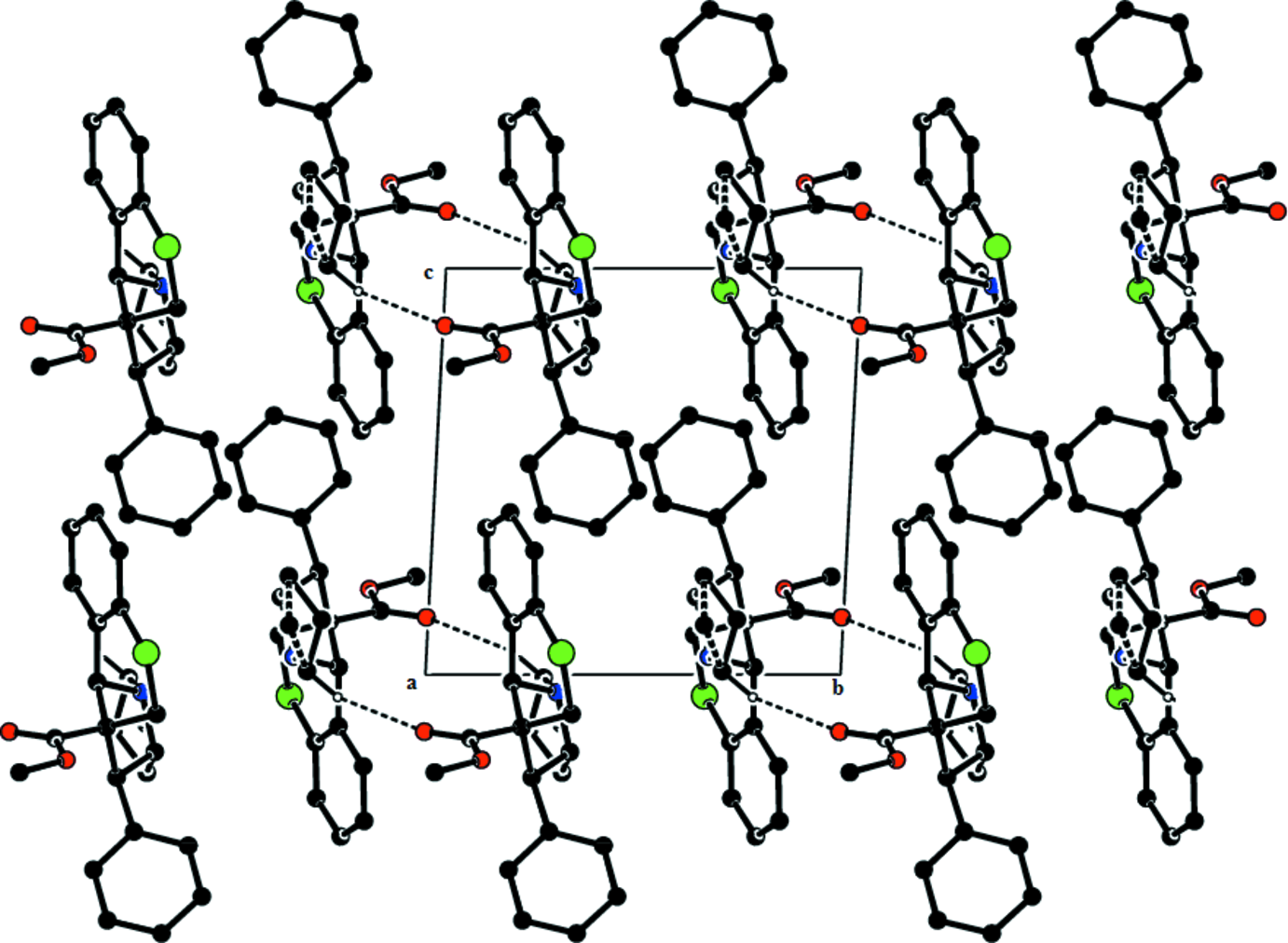

Supplement: Supplementary file 5 [file e-71-0o627-fig2.tif]
